# A new large-flowered species of *Andeimalva* (Malvaceae, Malvoideae) from Peru

**DOI:** 10.3897/phytokeys.110.29376

**Published:** 2018-11-05

**Authors:** Laurence J. Dorr, Carolina Romero-Hernández, Kenneth J. Wurdack

**Affiliations:** 1 Department of Botany, MRC-166, National Museum of Natural History, Smithsonian Institution, P.O. Box 37012, Washington, D.C. 20013-7012, USA National Museum of Natural History, Smithsonian Institution Washington United States of America; 2 Missouri Botanical Garden Herbarium, William L. Brown Center, P.O. Box 299, Saint Louis, MO 63166-0299, USA Missouri Botanical Garden Herbarium Saint Louis United States of America

**Keywords:** *
Andeimalva
*, Andes, Malvaceae, Malvoideae, Peru, phylogeny

## Abstract

*Andeimalvaperuviana* Dorr & C.Romero, **sp. nov.**, the third Peruvian endemic in a small genus of five species, is described and illustrated from a single collection made at high elevation on the eastern slopes of the Andes. Molecular phylogenetic analyses of nuclear ribosomal ITS sequence data resolve a group of northern species of *Andeimalva* found in Bolivia and Peru from the morphologically very different southern *A.chilensis*. The new species bears the largest flowers of any *Andeimalva* and is compared with Bolivian *A.mandonii*. A revised key to the genus is presented.

## Introduction

The genus *Andeimalva* J.A. Tate (Malvaceae, Malvoideae) was created to accommodate four species found in the Andes of South America from northern Peru to central Chile and includes three species previously placed in *Tarasa* Phil. and one in *Malacothamnus* Greene (Tate 2003). Krapovickas (1965) described two of these species, *T.machupicchensis* Krapov. and *T.spiciformis* Krapov., and allied them with a third, *T.mandonii* (Baker f.) Kearney, because he observed that the three differed from all other species of *Tarasa* in having entire lanceolate leaves more than three times as long as wide and awned mericarps with smooth (versus reticulate) sides. Despite the distinctiveness of this group, Krapovickas (1965) chose not to recognize it formally. The fourth species of *Andeimalva*, endemic to Chile, was long known as *M.chilensis* (Gay) Krapov. even though its placement in *Malacothamnus* was problematic based on its morphology, cytology, and geography (Bates 1963; Bates and Blanchard 1970; Tate and Simpson 2003). Tate (2003), combining molecular, cytological and morphological data, transferred these three species of *Tarasa* and one species of *Malacothamnus* to the newly described genus *Andeimalva*. The Chilean species of *Andeimalva* is anomalous morphologically in that it differs from the group recognized by Krapovickas (1965) in having suborbicular, slightly 3–5-lobed leaves and awnless mericarps. Nonetheless, molecular and cytological data indicate that it is closely related to the other three species found in Bolivia and Peru (Tate and Simpson 2003).

Recent floristic exploration in the northern Andes of Peru yielded a Malvaceae collection (*Vega Ocaña 419*) of uncertain initial generic affiliation. With further study and molecular phylogenetic analyses, we determined it to represent a new species of *Andeimalva*, which is described here as *A.peruviana* Dorr & C.Romero, and the third one endemic to Peru. We are amazed that a shrub with such conspicuous flowers is not known from earlier collections and can only speculate that either its range is very restricted in a botanically poorly explored area or it might simply have passed unnoticed due to a limited flowering period.

## Materials and methods

We sought molecular data both to confirm the generic identity of the new collection and to provide a phylogenetic context within the ITS (Internal Transcribed Spacer region of the nuclear ribosomal repeat) data set of Tate and Simpson (2003). Leaf tissue with abaxial trichomes scraped off was ground in a bead mill and extracted with the DNeasy Plant Mini Kit (Qiagen Inc., Valencia, California, USA) following an extended digestion with the AP1 buffer emended with 1.0 mg proteinase K (PCR grade solution, Roche Applied Science, Germany) and 1.8% v/v 2-mercaptoethanol. To reduce systemic contamination risk, all equipment was dedicated to “antique DNA” in a pre-PCR extraction laboratory and no other Malvales were co-extracted or amplified at the same time. Despite being a 2.5-year old collection with reasonably good preservation (i.e., retaining some color and alcohol treated), the resulting extraction only yielded highly degraded DNA (< 400 bp on an agarose test gel). Amplifications of ITS were attempted using a series of nested primers for decreasing amplicon sizes, and only a 302 bp fragment of ITS1 could be recovered with primers 1m (CGTAGGTGAACCTGCGGAAGGATC, newly modified from ITS1) and P2 (GCCRAGATATCCGTTGCCGAG; Cheng et al. 2016). Amplification reactions were conducted in a final volume of 15 µl with 1× Bioline reaction buffer, 8 mM dNTPs, 1.75 mM magnesium chloride, 2 nM of each primer, 0.1 mg/ml of bovine serum albumin, 0.025 U/µl of Biolase DNA polymerase (Bioline USA, Taunton, Massachusetts, U.S.A.), 1 M betaine, and 1 µl of DNA extraction. Sequencing was with BigDye Terminator v3.1 chemistry (Thermo Fisher Scientific, Waltham, Massachusetts) on an ABI 3730xl DNA Analyzer (Thermo Fisher Scientific). The new ITS1 sequence (GenBank MK044847, from *Vega Ocaña 419*) was manually aligned using a similarity criterion with the Tate and Simpson (2003) data set archived in TreeBASE (http://purl.org/phylo/treebase/phylows/matrix/TB2:M516). Small changes were made to the source matrix including converting mixed bases to ambiguity codes and improving the alignment within *Fuertesimalva* Fryxell (i.e., inconsistencies relative to positions 114–155 in our alignment), which reduced local branch lengths relative to prior analyses. The 53-tip matrix has an aligned length of 696 bp and relatively few, mostly small 1–4 bp indels (except *Fuertesimalva* spp. sharing a 42 bp deletion); we did not exclude any data in the analyses. We could not obtain any new plastid data and thus did not analyze the plastid data set of Tate and Simpson (2003), which the authors did not combine with ITS due to incongruence. Parsimony (MP) analyses were conducted with PAUP* 4.0a build 163 (Swofford 2002) using 1000 random addition replicates with TBR branch swapping and no search limits, and 1000 bootstrap (MP-BS) replicates, each with two random additions and a search limit of 10,000 trees per iteration (MulTrees = 10,000). Maximum likelihood (ML) analyses were conducted under a GTR + I + Γ model with GARLI 2.01 (Zwickl 2006) on CIPRES XSEDE (https://www.phylo.org/), using 100 replicates for optimal trees, and 1000 ML bootstrap replicates (ML-BS), each with two random additions and the automated stopping criterion.

### Data resources

The data underpinning the analyses reported in this paper are deposited in the Dryad Digital Repository at: https://doi.org/10.5061/dryad.44dm150

## Results

**Figure 1.**
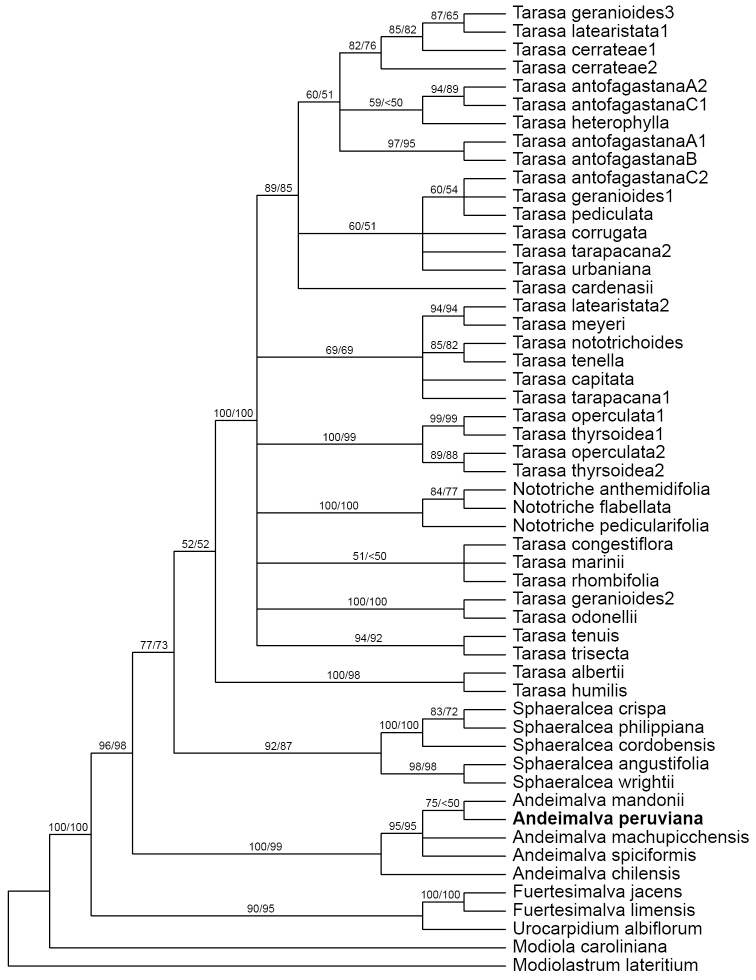
Maximum likelihood (ML) bootstrap 50% majority-rule consensus tree based on ITS data for *Andeimalva* and its Malvoideae relatives. ML/parsimony bootstrap values are indicated above branches.

Suspicions that *Vega Ocaña 419* belonged with *Andeimalva* were confirmed by molecular data where BLAST searches of our ITS1 sequence against GenBank yielded best matches with that genus. This affiliation was confirmed by phylogenetic analyses (Fig. 1) that placed it as strongly supported (BS 95–100) within a nested subclade of *Andeimalva*, although the exact sister relationship resolved with *A.mandonii* was weakly supported (ML-BS 75, MP-BS<50). Parsimony analysis yielded a single island with 180 trees (length = 346 steps, CI = 0.682 or 0.613, excluding uninformative characters; 128 parsimony informative characters) and a topology similar to prior phylogenies (i.e., Tate 2003; Tate and Simpson 2003). The topology of our ML tree (best score of 100 trees, -lnL = -3137.458) was also similar but not identical to any of the parsimony trees. There is a 3-bp deletion (loss of tandem GTT repeat at positions 280–282 in our alignment) only possessed by *A.chilensis* (Gay) J.A. Tate and *A.machupicchensis* (Krapov.) J.A. Tate, but this is not reflected in the recovered relationships and would not appear to be a structural synapomorphy.

### Taxonomy

#### Andeimalva peruviana

Taxon classificationPlantaeMalvalesMalvaceae

Dorr & C.Romero sp. nov.

urn:lsid:ipni.org:names:77191589-1

Figure 2

##### Type.

PERU. La Libertad: Bolivar, alpine bogs around Rio Negro and Pampa Uchulala along road to Bambamarca, 07°13'51"S, 077°38'21"W, 3750 m, 3 June 2015 (fl), *C. Vega Ocaña 419* with R.W. Bussmann, N. Paniagua Zambrana, F. Díaz Llajo & F. Díaz Vega (holotype: MO-2423556!; isotypes: HAO, US-01184179!, USM).

##### Diagnosis.

Differs from *Andeimalvamandonii* (Baker f.) Kearney in having larger stipules (13–15 × 5–8 mm versus 5–12 × 1–2 mm) that are broadly subulate (not filiform), and larger calyx lobes (1.7–2.1 × 1–1.8 cm versus 0.5–1 × 0.3–0.5 cm) and petals (3.5–4.5 × 2.5–3.2 cm versus 1.5–3 × 1.5–2 cm).

##### Description.

Shrubs, 1.5–3 m tall; young stems densely lanate and appearing white, glabrescent and dark brown to almost black in age. Stipules subulate with broader cordate base, 13–15 mm long, 5–8 mm wide, lanate with appressed stellate hairs, venation with 3 prominent parallel primaries running their length and up to 8 at base, persistent beyond life of leaf. Leaves simple, spirally arranged, petiolate, petioles 4–6 mm long, lanate; blades lanceolate to narrowly lanceolate, 5–8.2 cm long, 0.8–1.8 cm wide (lower leaf measurements due to serial size reduction toward the apex of flowering branches), base cordate, slightly asymmetric, apex acute, discolorous, drying dark green to brown above and whitish-tan below, bullate above with scattered sessile stellate hairs of varying sizes, the arms on the larger hairs ascending, midrib above densely pubescent and appearing white, densely lanate below with sessile stellate hairs and larger stalked, multi-rayed stellate hairs, the arms on the larger hairs spreading, stalks dark-colored, margin crenate-serrate, venation pinnate with 13–18 secondaries per side. Flowers solitary or in 2–3(–4)-flowered axillary or pseudo-terminal cymes, ca. 5–7 cm in diameter at anthesis, pedicellate, pedicels to 0.5 cm long; involucral bracts (2–)3, 1–1.8 cm long, 0.5–0.8 cm wide, inserted just below calyx, densely stellate pubescent, hairs near margin long-stalked and multi-rayed. Calyx lobes broadly deltoid, acuminate, slightly unequal in size, 1.7–2.1 cm long, 1–1.8 cm wide, pubescent within especially toward base, stellate-pubescent on the outside with small and large multi-rayed hairs. Petals obovate, unequal in size within the flower, 3.5–4.5 cm long, 2.5–3.2 cm wide, slightly to markedly asymmetric, deep mauve or dark magenta-purple, apex slightly undulate, unguiculate, glabrous except appressed simple hairs near base within and claw margins densely pubescent. Staminal column 2.8–3.2 cm long, sparingly pubescent throughout with simple hairs; anthers numerous (100+), 0.7–1.4 mm long, clustered at upper half of column; free portion of filaments (1–)1.6–5(–6) mm long. Carpels 10, cells uniovulate. Stigmas 20, capitate, scarcely exceeding the anthers at anthesis. Mericarps and seeds unknown.

**Figure 2.**
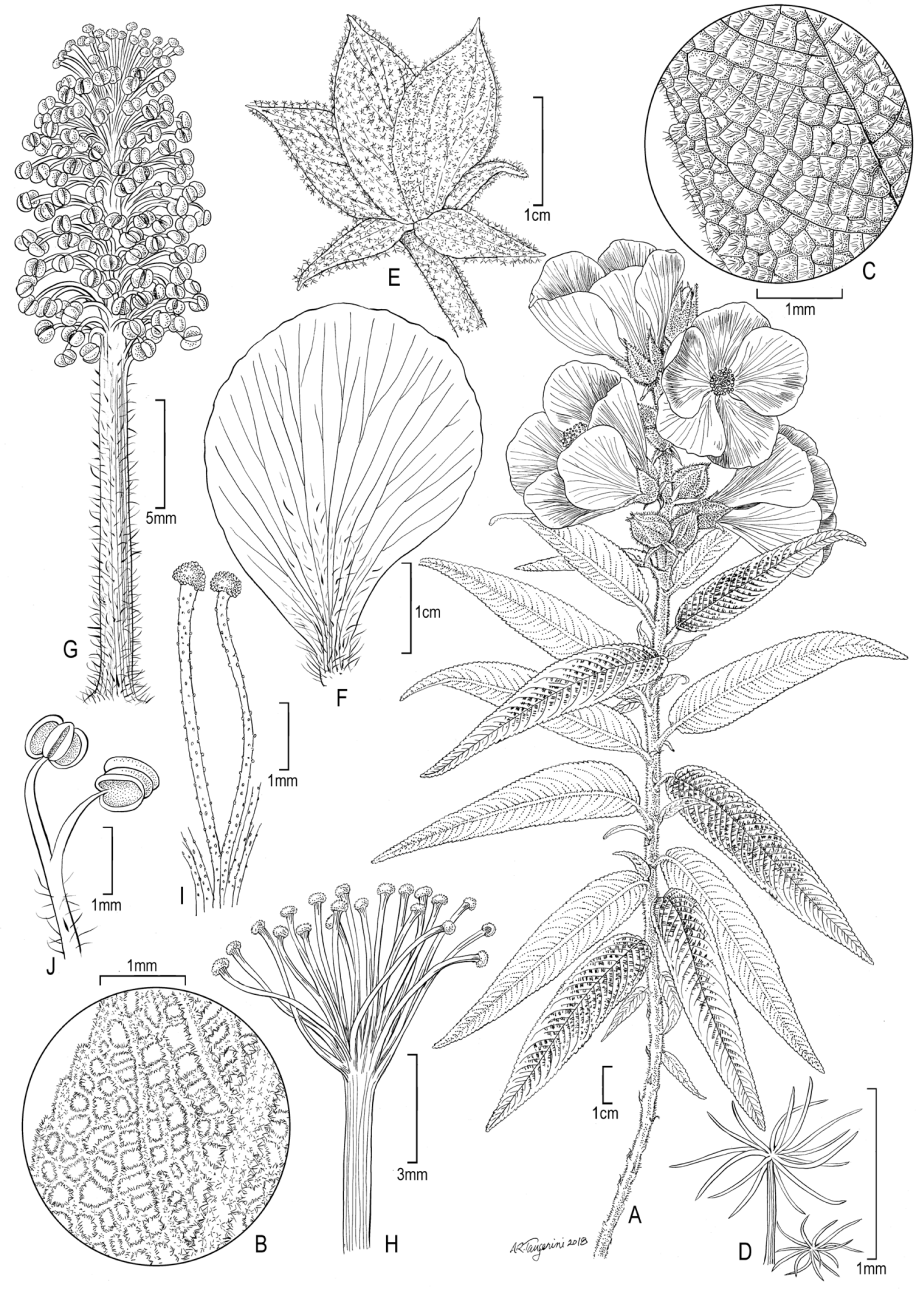
Illustration of *Andeimalvaperuviana*. **A** Habit **B** detail of leaf below **C** detail of leaf above: note bullate surface **D** stalked and sessile stellate trichomes from leaf blade below **E** involucral bracts subtending calyx (only 3 calyx lobes visible) **F** petal **G** staminal column: note stigmas scarcely exserted at apex **H** style with 20 capitate stigmas **I** detail showing two stigmas **J** detail showing two anthers (Source: *Vega Ocaña 419*, US).

##### Distribution and ecology.

At present, known only from the type locality where it occurs in alpine bogs at 3750 m.

On the label of the type specimen there is a slight discrepancy regarding the political subdivision for the locality. Google Earth Pro mapping (8 June 2011 imagery, https://www.google.com/earth/) places the coordinates cited on the label (presumably derived from a GPS device and for a logical locality near a road crossing of a wet drainage) in San Martín, and just over the eastern border delineated for La Libertad. However, we cannot verify how well Google Earth Pro finely draws political boundaries in relation to those that might appear on official government maps of such remote regions.

##### Etymology.

The specific epithet is derived from the name of the country (Peru) where the new species is found.

##### Preliminary conservation status.

Following the criteria and categories of IUCN (2017), *Andeimalvaperuviana* is given a preliminary status of Vulnerable (VU D2) due to population very small or restricted (area of occupancy < 20km^2^ and number of locations < 5). While the type of locality is quite remote and unexplored habitat occurs nearby, the general area has no protected regions and might be subject to habitat degradation from grazing or changes in hydrology. The latter includes human modification to its drainage system or climate change affecting montane precipitation.

## Discussion

Morphologically *Andeimalvaperuviana* appears to be closer to the three anomalous species of *Tarasa* first united by Krapovickas (1965) and later transferred to *Andeimalva* by Tate (2003), which share leaf morphology and mericarps with apical awns. *Andeimalvachilensis*, sister to the crown group (Fig. 1), has greater morphological divergence. However, its lack of awns relative to the other species of *Andeimalva* (uncertain in *A.peruviana*) may be an autapomorphic loss given that related genera such as *Tarasa* mostly possess awns (see Tate 2003). The chromosome number of our new species is unknown, but it would prove informative for generic affiliation. Optimal trees place *A.peruviana* as sister to *A.mandonii*, which has ML but not parsimony bootstrap support. This affinity is reflected in the morphology with shared generally larger floral features relative to the other species, but notably differs in stipule morphology (see Key below and Diagnosis). The large stipules of *A.peruviana* with prominent parallel major veins differ from those of the other species that are narrow in width and attachment.

Most species of *Andeimalva* are geographically well separated. However, *A.spiciformis* (Krapov.) J.A. Tate and *A.peruviana* are nearly sympatric on a gross scale with both occurring at high elevation in La Libertad, Peru. Nonetheless, the two species appear to be separated by altitude, hydrology, and a physical barrier. *Andeimalvaspiciformis* with its scattered distribution in northwestern Peru at 2400–3200 m, is known in La Libertad from a single collection (*A. López M. 1439*, US; see Tate 2003: 15) made at 2600 m. In contrast, the type locality of *A.peruviana*, about 70 km distant near the border of La Libertad, was made at 3750 m and is physically well separated by an east-west divide created by the deep valley of the Rio Marañon. The boggy habitat of *A.peruviana* in a region rich in small alpine lakes near the wet eastern side of the Andes generally appears much wetter than where *A.spiciformis* grows on drier mountain areas to the west where the Andean rainfall shadow has greater influence.

### Key to Species of *Andeimalva* (modified from Tate 2003)

**Table:** 

1	Leaves suborbicular, slightly 3–5-lobed; mericarps without an apical awn; central and southern Chile	*** A. chilensis ***
–	Leaves lanceolate, not lobed; mericarps with prominent apical awn (or character state unknown); Bolivia, Peru	**2**
2	Flowers in dense axillary glomerules; petals notched apically; mericarp awns 1–1.5 mm long; Peru	*** A. spiciformis ***
–	Flowers solitary or in few-flowered axillary cymes; petals not notched apically; mericarp awns 1.5–4 mm long (or character state unknown)	**3**
3	Staminal column < 2 mm long; calyx lobes 0.4–0.5 cm long; petals < 2 cm long; Peru	*** A. machupicchensis ***
–	Staminal column > 2 mm long; calyx lobes 0.5–2.1 cm long; petals 1.5–4.5 cm long	**4**
4	Petioles 3–12(–20) mm long; stipules 5–12 × 1–2 mm, filiform; staminal column 0.3–0.8 cm long; calyx lobes 0.5–1 cm long; petals 1.5– 3 cm long; Bolivia	*** A. mandonii ***
–	Petioles 4–6 mm long; stipules 13–15 × 5–8 mm, broadly subulate; staminal column 2.8– 3.2 cm long; calyx lobes 1.7–2.1 cm long; petals 3.5–4.5 cm long; Peru	*** A. peruviana ***

## Supplementary Material

XML Treatment for Andeimalva peruviana
